# Efficacy of 0.2 *μ*g/day fluocinolone acetonide implant (ILUVIEN) in eyes with diabetic macular edema and prior vitrectomy

**DOI:** 10.1038/eye.2016.303

**Published:** 2017-01-13

**Authors:** A Meireles, C Goldsmith, I El-Ghrably, A Erginay, M Habib, B Pessoa, J Coelho, T Patel, R Tadayoni, P Massin, J Atorf, A J Augustin

**Affiliations:** 1 Unit of Ophthalmology, CHP-Hospital Santo António, Porto, Portugal; 2 Unit of Ophthalmology, Universidade do Porto-Instituto Ciências Biomédicas Abel Salazar, Porto, Portugal; 3 Unit of Ophthalmology, James Paget University Hospital, Great Yarmouth, UK; 4 Unit of Ophthalmology, James Cook University Hospital, Middlesbrough, UK; 5 Unit of Ophthalmology, Lariboisiere University hospital, Paris, France; 6 Unit of Ophthalmology, Sunderland Eye Hospital, Sunderland, UK; 7 Department of Ophthalmology, Staedtisches Klinikum Karlsruhe, Karlsruhe, Germany

## Abstract

**Purpose:**

Limited data are available on the efficacy of the 0.2 *μ*g/day fluocinolone acetonide (FAc) implant in eyes with prior vitrectomy. Here, we present a collection of 26 vitrectomized eyes treated with the 0.2 *μ*g/day FAc implant.

**Methods:**

Retrospective study involving six centers from four European countries analyzing the safety and efficacy data from patients (26 eyes from 25 patients) with DME and a prior vitrectomy that had been treated with one 0.2 *μ*g/day FAc implant.

**Results:**

Prior intravitreal therapies included anti-VEGF (mean, 3.8 injections) and steroids (mean, 1.9 injections). Pars plana vitrectomy (PPV) was performed in these eyes primarily for abnormalities of vitreoretinal interface, followed by proliferative diabetic retinopathy and vitreous hemorrhage. The 0.2 *μ*g/day FAc implant was injected 24.2 months, on average, after PPV and the mean duration of follow-up after injection was 255 days (range, 90 to 759 days). The mean change in BCVA was +11.7 ETDRS letters (range, −19 to +40 letters; *P*<0.0004) and the mean change in central foveal thickness (CFT) was −233.5 *μ*m (range, −678 to 274 *μ*m; *P*<0.0001). The mean change in IOP from baseline at the last visit was +1.4 mm Hg (range, −9 to +8 mm Hg; *P*=0.0090). Eight eyes initiated or continued IOP lowering medications.

**Conclusions:**

These data suggest the 0.2 *μ*g/day FAc implant is effective in vitrectomized patients with an acceptable safety profile. Further studies are still required to confirm the current findings and to assess the effect of the 0.2 *μ*g/day FAc implant over a longer period of follow-up.

## Introduction

Vitrectomy is used as a treatment in DME eyes working to release vitreomacular traction, increasing the oxygenation of the retina and suppressing the diffusion of pro-permeability factors including IL- 6, vascular endothelial growth factor and intercellular adhesion molecule.^1^ The effect of this procedure on the duration of intravitreal therapy is still not properly understood. Indeed, a trial driven by the DRCR network, assessed the reduction of central retinal thickness during the first year of treatment with ranibizumab and reported a delayed efficacy in vitrectomized eyes, which could be related to drug clearance,^2^ despite beneficial long-term outcomes. However, there were no significant difference in effects, after adjustment for potential confounding effects and differences in baseline characteristics, in terms of visual acuity or central subfield thickness at any of the visits over the 3 year study period. Moreover, vitrectomized eyes required the same amount of intravitreal injections as non-vitrectomized eyes. The intraocular pharmacokinetics of ranibizumab and aflibercept in vitrectomized versus non-vitrectomized eyes is still unclear^3, 4, 5^ and would seem to be explained, in part, by the lack of agreement on the best animal model to be used as model for the human eye.^6, 7^

Steroids could be a useful therapeutic option in DME as sustained, chronic inflammation has been described in both animal and human models of diabetic retinopathy.^8^ A number of studies have assessed the effect of vitrectomy on the pharmacokinetics of steroids (mainly triamcinolone and dexamethasone implant). Indeed, animal models suggest that triamcinolone concentrations decrease up to 1.5 times more rapidly in vitrectomized eyes versus non-vitrectomized eyes.^7^ On the contrary, in a rabbit model studied during 1 month, the release kinetics of the dexamethasone implant is not believed to be impacted by prior vitrectomy.^9^ Some clinical results from 6 months trials with one single injection suggest that the kinetics of drug release from the dexamethasone implant would be the same in vitrectomized and non-vitrectomized patient eyes.^10, 11^ The peak pharmacokinetic profile is observed at 8 weeks and subsequently declines over a 6-month period.^10^ A recent paper reported opposite clinical results in 112 eyes with a significant increase in the required number of implants in vitrectomized eyes at month 12 and that no significantly differences were found in anatomical and functional outcomes.^12^ ILUVIEN (Alimera Sciences, Inc., Alpharetta, GA, USA) is a micro-implant, which is constituted by a non-biodegradable matrix made of polyamide. It contains 190 *μ*g of fluocinolone acetonide (FAc) released during 3 years (0.2 *μ*g of FAc per day released in the vitreous).^13^ The continuous release of FAc from this sustained drug delivery system could potentially counteract reported increases in drug diffusion, which increases drug clearance, in vitrectomized eyes and may even work to enhance the effect of vitrectomy in patients with DME.

In the FAME trial,^14^ vitrectomy was an exclusion criteria and so there is a paucity of clinical trial data following treatment with ILUVIEN. Hence, experience mainly comes from real-life clinical practice and here we present a collection of 26 vitrectomized eyes treated with one 0.2 *μ*g/day FAc implant.

## Materials and methods

This is a retrospective study involving patients treated with 0.2 *μ*g/day FAc implant in vitrectomized eyes from six centers. All centers had internal audits of their patients with vitrectomized and non-vitrectomized eyes. From those audits, all vitrectomized eyes were isolated and cumulated in a homogeneous database. Twenty-two patients were treated according to standard practices and three patients were previously included in a phase IV trial (identified on clinicaltrials.gov as NCT02472366).

Informed consent was obtained from all subjects at the injection of 0.2 *μ*g/day FAc implant. The main efficacy data collected were the change in ETDRS letter score. Nine eyes were prospectively evaluated with the decimal scale and were retrospectively converted to the ETDRS scale at the nearest value. Seventeen eyes were prospectively evaluated with the ETDRS scale and retrospectively collected for this study.

Central retinal thickness was evaluated on each site with spectral domain OCT. One center used Cirrus 1 from Carl Zeiss Meditech, Dublin, CA, USA. A second center used the 3D OCT-1 Maestro machine from Topcon Medical Systems, Inc. Oakland, NJ, USA. Four centers used Spectralis (3 OCT/1 HRA) from Heidelberg Engineering, Vista, CA, USA. As normal range vary with OCT machines, anatomical results are described according to the mean change.

IOP values were also reported from each center with examinations according to local practices.

### Statistical analysis

The statistical method used to test to see whether the mean change from baseline to the last observation was different from zero was a one-sample *t*-test. This parametric statistical test fits with the number of eyes studied (*n*>30). For the BCVA gain and for the change in central retina thickness, units reported are the mean value and the SD. Statistical analysis was performed with the global number of 26 patient eyes. IOP change was analyzed in the same way. The software used to generate the results and to perform the statistical test was SAS version 9.1.3 (Cary, NC, USA).

## Results

A total of 26 vitrectomized eyes from 25 patients were treated with one injection of 0.2 *μ*g/day FAc implant with a mean follow-up of 8.5 months. Baseline characteristics are described in Table 1. All patients were treated for DME. All eyes except one were pseudophakic. Fifty four percent of the patients had more than one indication for the PPV. The main indication for PPV were abnormalities of the vitreoretinal interface which contained 7 macular pucker, 9 tractional retinal detachments, and 2 fibrovascular proliferations. The mean time between PPV and the injection of 0.2 *μ*g/day FAc implant was 24.2 months with a range between 3.6 and 73.3 months. Two eyes were naive of prior intravitreal treatment before the injection of 0.2 *μ*g/day FAc implant. Fifteen eyes had prior anti-VEGFs in combination with steroids, five had anti-VEGFs alone and four had steroids alone. The main anti-VEGF prescribed as prior treatment was ranibizumab. For the steroid class, only triamcinolone was reported with doses from 4 to 25 mg. The initial IOP response to triamcinolone is unknown in 5 patients, is mild or good in 13 patients and 1 patient had a description of persistently raised IOP after his first injection of triamcinolone.

**Figure 1:**
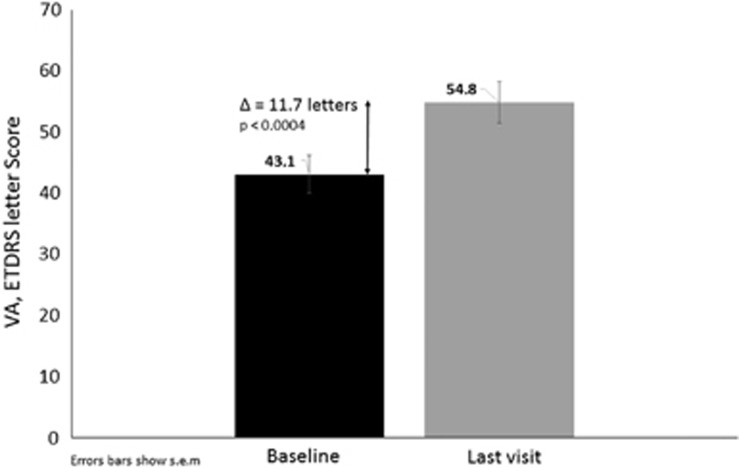
Change in visual acuity (VA; ETDRS letter score) from baseline to the last visit.

**Figure 2:**
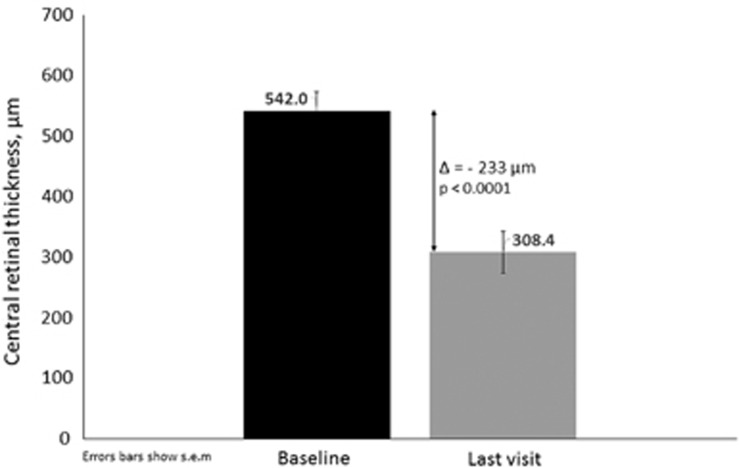
Change in central retinal thickness from baseline to the last visit.

**Figure 3:**
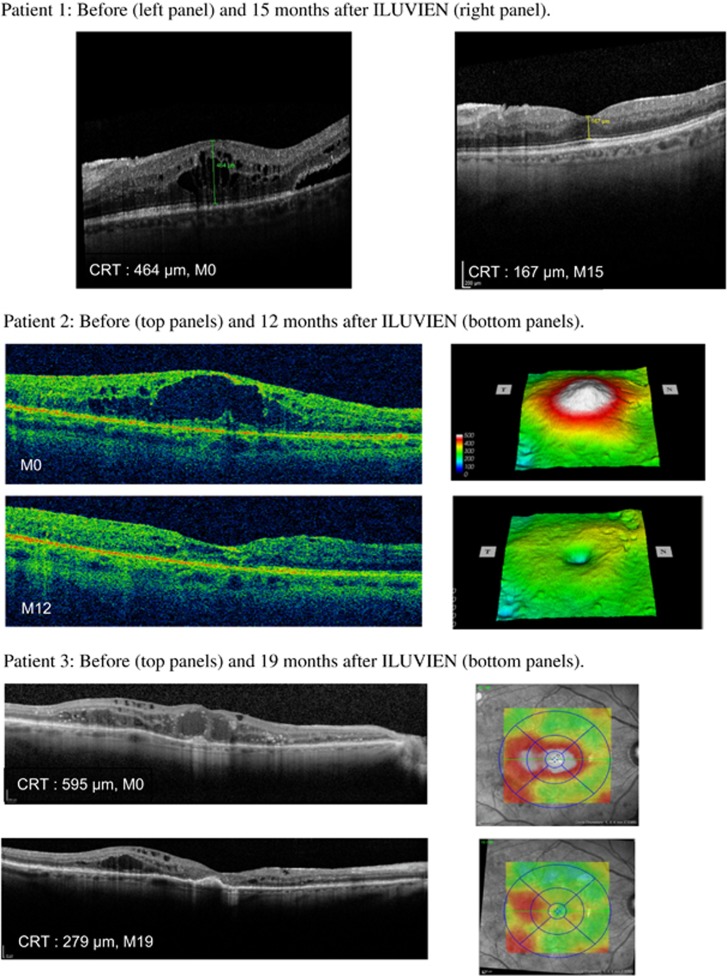
Anatomical response. Optical coherence tomography images from three patients.

Main efficacy results are described in Figures 1 and 2, and the individual results from 3 of the 25 patients are shown in Figure 3. There is a significant (*P*<0,0004) mean gain of ~12 ETDRS letters (range, −19 to +40) from baseline to the last visit in the 26 vitrectomized eyes. At the baseline, 8% of the eyes reached at least 70 letters (the threshold for driving in Europe) compared with 27% at the last visit. Of the 26 eyes, 17 had a gain of at least 5 ETDRS letters at the last visit of follow-up and 5 had stable vision (a gain between 0 and 4 letters). Meaning that with a single 0.2 *μ*g/day FAc implant, 84.6% of eyes gained or maintained vision.

The average reduction of the CRT at the last visit was −233 *μ*m (range, −678 to 274 *μ*m, *P*<0.0001). At this visit, 84.6% (*n*=22/26) of the patient eyes displayed any decrease of their edema. In 80.8% (*n*=21/26) of the patient eyes this decrease was at least 100 *μ*m. Note that 46.1% of patient eyes gained at least 5 letters and had a reduction in edema of at least 100 *μ*m at their final visit. No eyes had a loss of BCVA with an associated increase of retinal thickness at the last visit.

In this cohort, the duration of follow-up did not seem to impact the results. Indeed, in thirteen patients with a follow-up shorter than the median of 180 days, 84.6% (*n*=11/13) of patient eyes had a gain of vision compared with 84.6% (*n*=11/13) of patient eyes with a follow-up period >180 days. In terms of anatomical response, 76.9% of patient eyes below the average follow-up time had a decrease in retinal thickness compared with 92.3% of patient eyes with a follow-up period >180 days. The longest follow-up period was 759 days from a patient who experienced a gain of 24 letters and a decrease in retinal thickness of 231 microns with a single injection of the 0.2 *μ*g/day FAc implant. Three patients had associated injections during their follow-up (the first patient had 3 ranibizumab injections followed by 2 aflibercept injections in a context of associated renal disease with possible amyloid; the second patient had 2 injections of aflibercept; and, the third one had one aflibercept injection on the same day as phacoemulsification).

IOP related events after injection of 0.2 *μ*g/day FAc implant are summarized in Table 2. IOP-lowering medications were initiated in 30.8% of patient eyes. Two anterior migrations of the 0.2 *μ*g/day FAc implant were reported in patients with a previous capsular tear and were surgically reinserted in the vitreous without further complications.

## Discussion

This is the first multi-national, multi-center center study to report the outcome following a single injection of the 0.2 *μ*g/day FAc implant in 26 previously vitrectomized eyes with DME that had previously been treated with anti-VEGFs and/or steroids. Significant changes were found with a 43.0% mean decrease of CRT and an average gain in visual acuity of more than two ETDRS lines over a mean follow-up period of 8.5 months. These findings from real-life practice are important in guiding the use of steroids in vitrectomized eyes in patients with DME. The current findings highlight the lack of consensus on the management of this group of patients and an opportunity to develop a treatment pathway.

The current results extend the current knowledge concerning the use of a single 0.2 *μ*g/day FAc implant in vitrectomized eyes. Results support those previously reported by Elaraoud *et al*^15^ in 5 vitrectomized eyes from a cohort of 22 pseudophakic eyes. As well as having fewer eyes, that study was an audit after 3 months to assess the early outcomes from three UK clinics (Wolverhampton Eye Infirmary, Queen Elizabeth University Hospital and the Royal Hallamshire Hospital). This study reported the results from 5 eyes that had undergone prior vitrectomy and visual acuity was shown to improve by +7.2 letters (range: 0 to +14) and that CRT decreased −176.8 (range: −714 to +385) after 3 months. Four of five eyes showed both a reduction in CRT with improved visual acuity but one patient had reduced CRT (-345) with no gain in visual acuity. To date, no further data have been published on these vitrectomized eyes and the current study has merit as it has ≥3 months follow-up in 26 patient eyes.

In the current study, all patients received a single ILUVIEN injection during the studied period. This simple regimen administration is particularly interesting in diabetics. In fact, more than half of patients who receive intravitreal injections for DME spend up to 20 h over a 6-month period with health care professionals for the management of diabetes and associated complications.^16^ Kiss *et al*^17^ have recently shown a linear correlation between the number of anti-VEGF injections and efficacy outcomes. And other studies suggest there are fewer anti-VEGF injections in real-life practice^18^ compared with controlled clinical trials^19^ (3.7−4.7 injections per year *vs* 7.0−12.2 injections per year, respectively). This suggests that poor compliance may be the major hurdle for optimal efficacy outcomes in real-life clinical practice. The same is also true for the dexamethasone implants with randomized clinical trials reporting variable administration regimes and changing from one fixed dose every 6 months to a fixed dose regime every 5 months or even a PRN regimen.^20^ The reinjection interval could also be difficult to predict in vitrectomized eyes if the number of dexamethasone implants used over a 12 months period differs between vitrectomized and non-vitrectomized, which has been reported by the PACORES group recently (ie, 3 *vs* 1 dexamethasone implant every 12 months, *P*<0.001).^26^

A potential issue with injection of implants in vitrectomized eyes is the impact of the device with the retina structure as the implant is no more slowed down by a less visceous vitreous. Moysidis *et al*^27^ have shown this phenomenon in animal model with dexamethasone implants showing a collision in 3 out of 4 vitrectomized eyes compared with 0 in non-vitrectomized eyes. The potential clinical relevance of this impact of the implant on the retina is not yet defined. We don't expect the same issue with the 0.2 *μ*g/day FAc implant which has no spring and which is deposed in the back of the retina.

The current study showed that the 0.2 *μ*g/day FAc implant migrated to the anterior chamber in two eyes due to the presence of a capsular tear. However, a novel technique has recently been published to show that the migrated implant can be repositioned and reinsertion into the vitreous cavity without compromising implant integrity.^28^ To date there have been four reported cases of the 0.2 *μ*g/day FAc implant migrating into the anterior chamber^28, 29^ and these have related to the use of the 0.2 *μ*g/day FAc implant in vitrectomized eyes and complicated cataract surgery where one patient had the implant removed due to recurrence of the migration. Hence, caution is required in vitrectomized eyes where there is a disruption in the posterior capsule.

The main limitations of this study relate to the collection and reporting of retrospective data with a relatively small number of patients and short period of follow-up post-therapy with ILUVIEN. A further limitation is the reporting of non-standardized data, which were collected in real-life clinical practice. However, sustained, and statistically significant, therapeutic effects were observed in this small cohort over a 2-year period and seem to be consistent with results reported in larger randomized controlled clinical trials.

Future studies should consider whether the difference in biochemical composition of the vitreous in vitrectomized eyes and non-vitrectomized eyes affects the pharmacokinetic profile of ILUVIEN and consequently its efficacy. Another area of interest is to assess whether PPV enhances the performance of ILUVIEN when performed after ILUVIEN, and therefore at any stage in the disease process, as has been described by Kumar *et al.*^30^

This paper describes the most clinically significant cohort of vitrectomized eyes treated with a 0.2 *μ*g/day FAc implant. Results show there were statistically significant improvements in visual acuity and a concurrent decrease of the macular edema. The current study is important as the FAME trial excluded patient eyes with prior vitrectomy and so the real-life data are now needed. This lack of the data and clinical need mean that there is no consensus on how to use DME therapies to manage vitrectomized eyes with DME, and there is no treatment pathway on to guide physicians. Hence, current real-life practice data are revealing how these eyes are being managed now but also informing future best practice.

## Conclusions

It is estimated that around 20% of patients with DME have had prior PPV before being treated with a 0.2 *μ*g/day FAc implant. The current results show continuous therapy delivered with a single implant, lasting up to 8.5 months in this case, can lead to clinically relevant outcomes in both visual acuity and the reduction of retinal edema with a manageable safety profile. PPV is frequently indicated in late complications of diabetic retinal abnormalities, however, considering the present results, ILUVIEN could be considered earlier in the DME disease process if an insufficient response has been observed in patients after the first loading dose of an anti-VEGF. The single injection technology would also avoid the need for repeated injections and help to mitigate poor patient compliance and associated treatment anxiety. Other studies of vitrectomized eyes treated by 0.2 *μ*g/day FAc implant have to be conducted to confirm our data and to confirm the effects over a longer follow-up period.


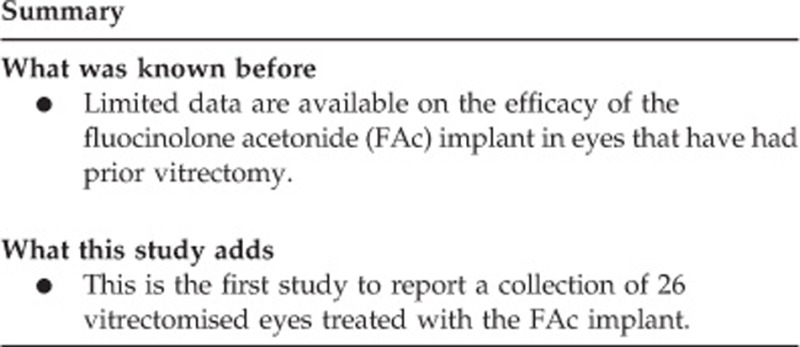


## Figures and Tables

**Table 1:** Baseline characteristics of the patients at the injection of FAc

*Parameter*	*Results (*n *or mean*±*SD)*
Number of eyes/patients	26/25
Age	67.0 years±11.8
Male	19 (76%)
Diabetic macular edema (DME)	26 (100%)
Time since diagnosis of DME	44.1 months±25.6
Disease related to DME	Diabetes 25 (100%) Type I, 4 (16%) Type II, 15 (60%) Not reported, 6 (24%)
Time since PPV	24.2 months±17.4
Reason for PPV	Abnormalities of vitreoretinal interface 20 (77%) Proliferative diabetic retinopathy 12 (46%) Vitreous hemorrhage 11 (42%) Others 1 (4%)
Pseudophakic eyes	25 (96%)
*Prior therapy*	26 (100%)
Grid/ focal laser	12 (46%)
Anti-VEGF	20 (77%)
Steroids	19 (73%)
Prior panretinal photocoagulation	22 (85%)
BCVA	43.1±16 ETDRS letters
Central retinal thickness	542.0 *μ*m±245
Intraocular pressure	14.9 mm Hg±3

**Table 2:** IOP events and IOP management

*Parameter*	*Mean±SD*
*IOP [mm Hg]*	
Baseline	14.9±3
Mean maximal value over time	20.3±9
Change from the baseline at the last visit, *P*-value	+1.4±4, *P*=0.009
	
*Management*	
Lowering IOP medication, n (%)	8 (31%)
Lowering IOP surgery, n (%)	0 (0%)
